# Design of Monolithic 2D Optical Phased Arrays Heterogeneously Integrated with On-Chip Laser Arrays Based on SOI Photonic Platform

**DOI:** 10.3390/mi13122117

**Published:** 2022-11-30

**Authors:** Jian Yue, Anqi Cui, Fei Wang, Lei Han, Jinguo Dai, Xiangyi Sun, Hang Lin, Chunxue Wang, Changming Chen, Daming Zhang

**Affiliations:** 1 State Key Laboratory of Integrated Optoelectronics, College of Electronic Science and Engineering, Jilin University, Changchun 130012, China; 2 China Xi’an Satellite Control Center, Xi’an 710000, China

**Keywords:** optical phased array, on-chip laser arrays, EO switching/shifting array, Bragg waveguide grating antenna array

## Abstract

In this work, heterogeneous integration of both two-dimensional (2D) optical phased arrays (OPAs) and on-chip laser arrays based on a silicon photonic platform is proposed. The tunable multi-quantum-well (MQW) laser arrays, active switching/shifting arrays, and grating antenna arrays are used in the OPA module to realize 2D spatial beam scanning. The 2D OPA chip is composed of four main parts: (1) tunable MQW laser array emitting light signals in the range of 1480–1600 nm wavelengths; (2) electro-optic (EO) switch array for selecting the desired signal light from the on-chip laser array; (3) EO phase-shifter array for holding a fixed phase difference for the uniform amplitude of specific optical signal; and (4) Bragg waveguide grating antenna array for controlling beamforming. By optimizing the overall performances of the 2D OPA chip, a large steering range of 88.4° × 18° is realized by tuning both the phase and the wavelength for each antenna. In contrast to the traditional thermo-optic LIDAR chip with an external light source, the overall footprint of the 2D OPA chip can be limited to 8 mm × 3 mm, and the modulation rate can be 2.5 ps. The ultra-compact 2D OPA assembling with on-chip tunable laser arrays using hybrid integration could result in the application of a high-density, high-speed, and high-precision lidar system in the future.

## 1. Introduction

The silicon-on-insulator (SOI) photonic platform [1,2] has attracted tremendous attention owing to its mature processing and scalable compatibility of the complementary metal oxide semiconductor (CMOS) technique. In particular, the monolithic optical phased arrays (OPAs) based on SOI photonic integrated circuits (PICs), with the merits of low cost, small footprint, and high stability, could become an essential component for the light detection and ranging (LIDAR) module [3,4,5]. Currently, for achieving two-dimensional (2D) beam steering, there are two main ways, including tuning only the phase [6,7] or tuning both the wavelength and the phase [8,9]. Compared to steering with only the phase, a larger scanning angle range and higher operating bandwidth could be realized by steering both the wavelength and phase. Meanwhile, superior to butt-coupling typed light sources [10,11,12,13], hybrid integration by bonding active III–V dies onto a SOI platform [14,15,16,17,18] could provide ultra-compact and high-quality on-chip lasers with arrayed lasing wavelength emission. The heterogeneous integration of on-chip semiconductor lasers with SOI PICs will be more suitable for realizing large-scale, dense-integration, and energy-efficient solid-state LIDAR system application. Furthermore, in contrast to Si-based thermo-optic (TO) modulation [19,20], Si-based electro-optic (EO) wavelength-selective switches and phase shifters [21] have significant features of high-speed response and low power consumption. The EO-type tuning elements could improve the dynamic performance of active SOI PICs for OPA chips.

In this paper, monolithic 2D optical phased arrays heterogeneously integrated with on-chip laser arrays are proposed based on the SOI photonic platform. The four main components of the compact system-on-chip multi-quantum-well (MQW) laser arrays, EO switching array, EO phase-shifter array, and Bragg waveguide grating antenna array are designed and simulated. The performances of the 2D OPA chip are optimized. A large steering range is realized by tuning the phase and the wavelength of each antenna. The overall fabrication process for realizing the OPA photonic chip is mature. In contrast to the traditional thermo-optic LIDAR chip with an external light source, the overall size of the 2D OPA chip can be limited within 1 cm^2^, and the modulation rate can be increased from -μs to -ps. The ultra-compact 2D OPA assembling with on-chip tunable laser arrays on a silicon photonic platform is suitable for achieving a high-integration, high-speed, and high-precision lidar system.

## 2. Design and Simulation

### 2.1. Overall Description of the 2D OPAs Integrated with On-Chip Laser Arrays

**Figure 1:**
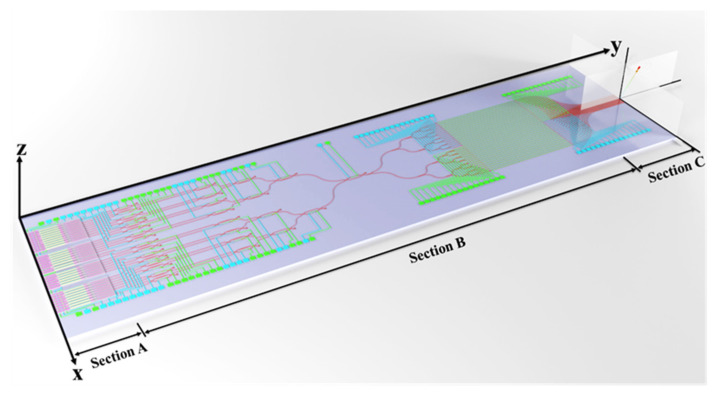
Schematic diagram of the proposed OPA showing the main parts.

The monolithic 2D OPA module based on the proposed SOI photonic platform is composed of on-chip III–V heterogeneous-integration MQW laser arrays (Section A), EO switching/phased shifter arrays (Section B), and grating emitter arrays (Section C). The schematic structure of the entire 2D OPA integrated module is shown as Figure 1.

In Section A, 32 channel active on-chip heterogeneous-integration laser arrays depending on the Fabry-Pérot (F-P) cavity are divided into four groups based on the different thickness of the InGaAs/InGaAsP MQW structures. The MQW laser arrays from the four groups could be thermally tuned to generate the wavelength signals from 1480 to 1600 nm (from S to L bands), with wavelength spacing of approximately 4 nm. The light signals from the MQW laser arrays are propagated into the passive InP connecting waveguide using a taper structure, which could guarantee fundamental mode transmission, and imported into bottom passive silicon waveguides through interlayer coupling technique. Then, 32 single-mode wavelength signals are transmitted into Section B. In Section B, 32 × 1 EO switching arrays are cascaded with 1 × 32 channel EO phased shifter arrays. The wavelength-channel-selection function is achieved using 32 × 1 EO switching arrays. The selected wavelength signal could be split up into 32 channels, and wavelength-phase shifting-difference control for the 32 channels is accomplished by 1 × 32 channel EO phase-shifter arrays. The key role is to regulate the signal wavelengths and modulate the optical phase based on the silicon photonic platform. In Section C, 32 channel Si waveguide grating emitters as the antenna arrays of OPAs are defined. By tuning the optical wavelength and phase, the 2D beam steering performance of OPAs could be realized for X–Y plane direction scanning. In this design, ultra-compact, high-speed, and dense-integration 2D OPAs with on-chip optical sources are built on the SOI photonic platform.

### 2.2. Design for On-Chip Heterogeneous-Integration MQW Laser Arrays (Section A)

**Figure 2:**
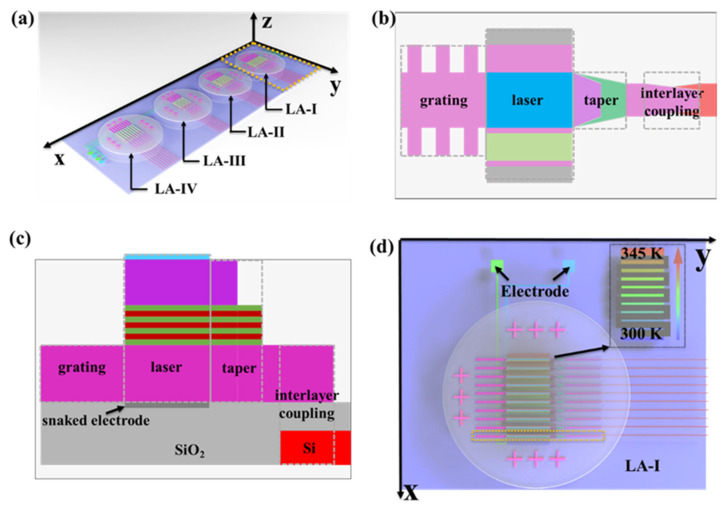
The structure of integrated laser arrays on the chip. (**a**) The overall structure of laser arrays. (**b**) The top view of the F-P MQW laser. (**c**) The cross-section of the F-P MQW laser. (**d**) The temperature-control electrode layout scheme of LA-I.

As shown in Figure 2a, four InP wafers with epitaxial growth [22,23] of different thickness MQW structures could be loaded on the Si photonic chip via the wafer-bonding technique [24,25]. The parameters designed for on-chip heterogeneous-integration InGaAs/InGaAsP (Q = 1.25 μm) MQW laser arrays [26] into four groups could be achieved with the standard semiconductor fabricating process (provided by Prof. Sengtiong Ho’s Laboratory of Electrical and Computer Engineering Department of Northwestern University in America) by including 10, 6, 8, and 8 laser units for each group, respectively. The F-P cavity with the distributed Bragg reflection (DBR) of a laser unit is defined in Figure 2b,c. The emitting wavelength from the MQW laser could be transmitted into the InP waveguide by tapering the structure, and then into the bottom silicon waveguide via the interlayer coupling architecture. In the 1480–1600 nm wavelength band, varied emitting wavelength regions are set on the basis of the different thickness of MQW structures as laser arrays (LA) -I, -II, -III, and -IV. For achieving emitting wavelengths with equal spacing, bottom metal controlling thermal heaters are designed on the upper surface of the SiO_2_ layer, where the InP wafers bonded, as shown in Figure 2c. As given in Figure 2d, the snaked electrodes with different widths could modulate the laser arrays considering the thermo-optic effect by changing the working temperature. Using LaserMOD and BeamPROP of the Rsoft software, the performances of on-chip III–V heterogeneous integration MQW laser arrays (Section A) are analyzed and simulated with the beam propagation method (BPM) and Minilas-II algorithm, respectively.

**Figure 3:**
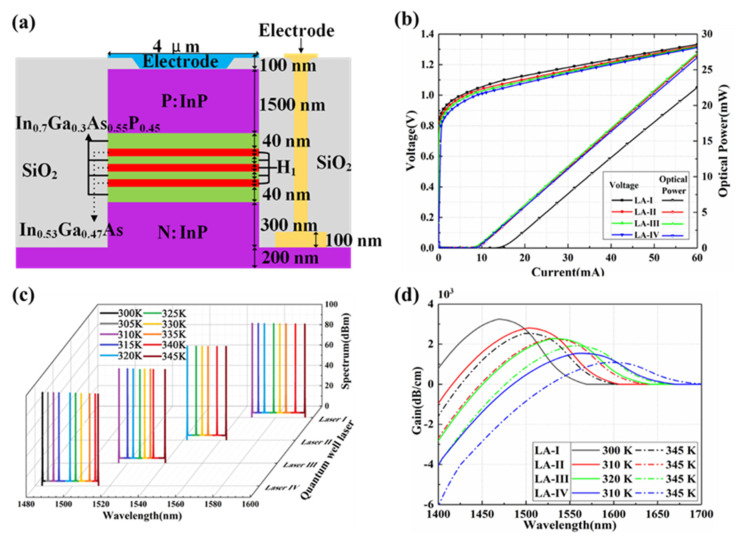
(**a**) The cross-section of the FP quantum well laser. (**b**) I–V curves of the four types of quantum well lasers. (**c**) Effects of temperature on the emission spectra. (**d**) Gain spectra of the four types of quantum well lasers.

The cross-sectional structure of the MQW laser is illustrated in detail in Figure 3a. Three layers of In_0.53_Ga_0.47_As/In_0.3_Ga_0.7_As_0.55_P_0.45_ quantum wells are defined. The thickness of a single In_0.53_Ga_0.47_As quantum well and a single In_0.3_Ga_0.7_As_0.55_P_0.45_ barrier is equal to *H_1_* (6 nm for LA-I, 7 nm for LA-II, 8 nm for LA-III, and 10 nm for LA-IV). The thickness of the upper and lower SCH barriers is set at 40 nm. The 1500 nm-thick P-type InP top cladding and 500 nm-thick N-type InP bottom cladding serve as the P- and N-ohmic contact layers, respectively. The detailed parameters such as the doping type, thickness, refractive index at 1550 nm wavelength, and the carrier concentration for each layer of the MQW laser structure are given in Table 1 [26]. The I–V characteristic curves of the MQW laser arrays at room temperature (300 K) are simulated and shown in Figure 3b. The operating voltage is approximately 1.1 V, and the threshold current is less than 15 mA. The driving electrical consumption of the device is low. As observed in Figure 3c, when the value of *H_1_* is increased from LA-I to -IV, the emission spectra of the MQW laser arrays are red-shifted at room temperature. The interval value of the emitting peak wavelength from 1480 to 1600 nm for the adjacent LA group is approximately 30 nm. By adjusting the working temperature of the laser unit for each LA group using the bottom snaked electrode heaters properly, the emitting wavelength spacing of the adjacent laser unit for each LA group is equal to 3.75 nm. Moreover, the optical power of the laser is more than 80 dBm. The related featured distribution of emitting wavelengths for LA-I, -II, -III, and -IV is shown in Figure 3d. When the working temperature is in the range of 300 K–345 K, the gain value of the laser unit could remain more than 1 × 10^3^ dB/cm. Based on heterogeneous integration, the on-chip III–V MQW laser arrays proposed could provide large scanning bandwidth and high scanning precision for wavelength and phase steering. It is suitable for achieving ultra-compact high-performance OPA arrays based on the SOI photonic platform.

**Figure 4:**
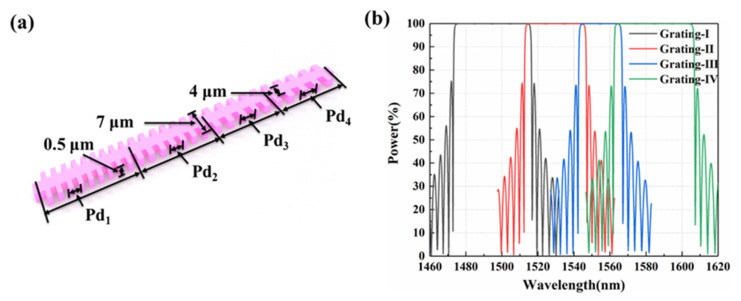
Reflection gratings: (**a**) unit structure; (**b**) reflection spectra.

To enhance the emitting efficiency of the lasing wavelengths, the cascaded sampling grating DBR structure for the F-P cavity is designed for the different LA groups, as shown in Figure 4a. The width, ridge height, and thickness of the waveguide grating are 4.0, 1.5, and 0.5 μm, respectively. The duty cycle is defined as 50%. The length of the initial grating period is set as Pd. As given in Table 2, for varied emitting lasing wavelength regions of the different LA groups, the number N of the waveguide gratings with Pd +NδPd is cascaded for realizing the DBR architecture. The reflecting spectra are given in Figure 4b. The reflected power of the four cascaded reflection gratings is more than 99% in the wavelength range of 1480–1600 nm.

**Figure 5:**
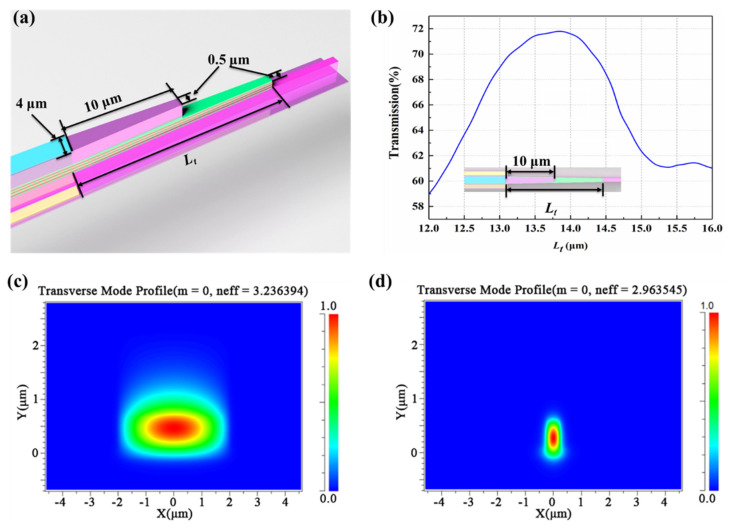
(**a**) The stepped trapezoidal coupling structure between the F-P quantum well laser and InP waveguide. (**b**) The relationship between transmission and the length (*L_t_*) of the trapezoidal quantum well and InP waveguide. (**c**) Cross-section optical field distribution of the input region. (**d**) Cross-section optical field distribution of the output InP waveguide.

After that, the emitting signal light from the MQW laser is coupled into the N-InP dielectric waveguide using the taper structure as shown in Figure 5a. The stepping taper coupling structure is composed of two parts: taper-1 and taper-2. The initial width of taper-1 and taper-2 is 4 µm. The length of taper-1 with InP top cladding is defined as 10 µm and that of taper-2 without InP top cladding is set as *L_t_*. The width of the tip for both taper-1 and taper-2 is set as 0.5 µm. The relationship between coupling efficiency and *L_t_* is given in Figure 5b. When *L_t_* is 13.8 µm, the maximum coupling efficiency can come to 71.8%. The coupling optical field distribution of taper-1 and taper-2 and the dielectric waveguide is shown in Figure 5c and d, respectively. The stepping taper coupling structure could guarantee single-mode propagation of emitting lasing wavelength and suppress mode energy loss by optimizing the coupling waveguide structure.

**Figure 6:**
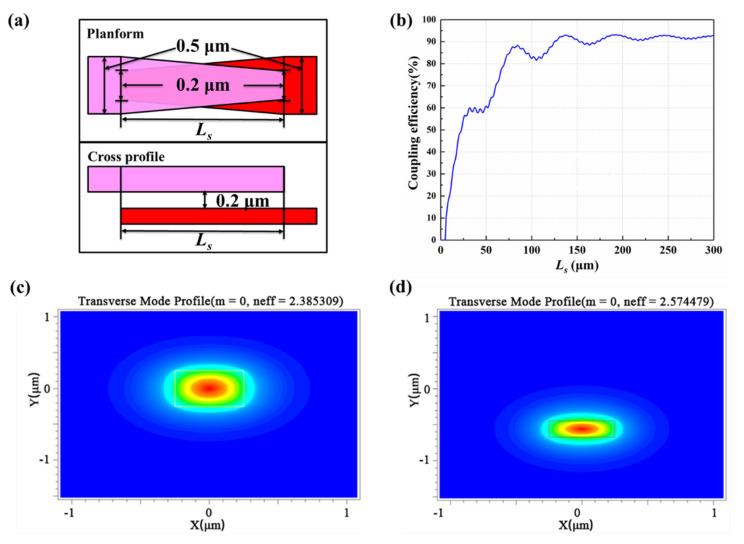
(**a**) The bidirectional taper structure between the InP and Si waveguide. (**b**) The relationship between the transmission and the length (*L_s_*) of the bidirectional taper structure. The cross-section optical field distribution of (**c**) the InP waveguide and (**d**) the Si waveguide.

To make the lasing signal light from the N-InP dielectric waveguide into the Si waveguide efficiently, interlayer taper coupling structures for both InP and Si waveguides are designed, as given in Figure 6a. The coupling length between the InP and Si waveguides is set as *L_s_*, and the SiO_2_ layer gap between the InP and Si waveguides is set as 0.2 μm. The fabrication tolerance relies on the 180 nm silicon photonic multi-project-wafer (MPW) process provided by the Institute of Microelectronics of Chinese Academy of Sciences (IMECAS). The initial width and ending tip for the InP and Si waveguide are the same, at 0.5 and 0.2 µm, respectively. As shown in Figure 6b, when *L_s_* is defined as 137 µm, the maximum coupling efficiency of 92% could be realized. The coupling optical field distribution of the InP and Si waveguide is simulated in Figure 6c and d, respectively. Single-mode transmission of the optical signal can be achieved.

### 2.3. Design for the EO Switch Array and Phase-Shifter Array (Section B)

**Figure 7:**
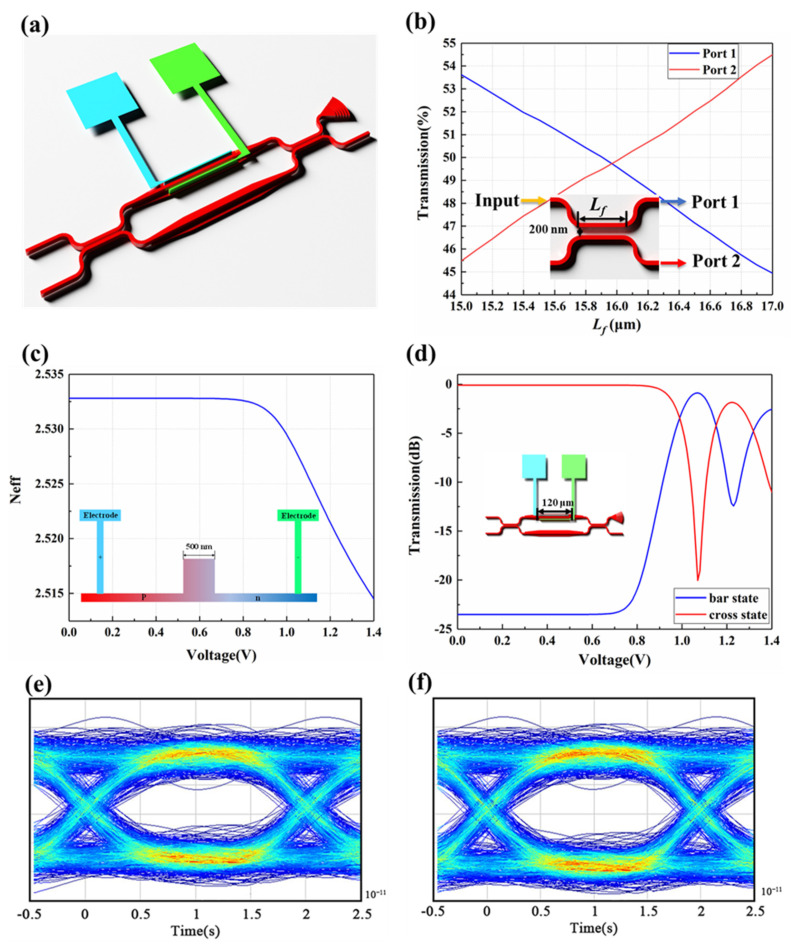
(**a**) Switching unit structure. (**b**) The relationship between coupling length (*L_f_*) and transmission. The relationship between voltage and (**c**) effective refractive index (*Neff*) and (**d**) transmission. The eye diagram at the wavelength of (**e**) 1480 nm and (**f**) 1600 nm.

In Section B, the 32 × 1 SOI waveguide EO switch array is designed to select the desired signal wavelength to be sent to the 1 × 32 SOI waveguide EO phase-shifter array. The defining data of the silicon photonic integrated circuits are from the standardized 180 nm silicon photonic multi-project-wafer (MPW) process, provided by the Institute of Microelectronics of Chinese Academy of Sciences (IMECAS). Using the Lumerical Software, the performances of the 32 × 1 SOI waveguide EO switch array and the 1 × 32 SOI waveguide EO phase-shifter array (Section B) are analyzed and simulated using the finite-difference time-domain (FDTD) method. The switching unit is composed of two 3 dB directional coupling (DC) couplers and Mach-Zehnder interferometer (MZI) modulation arms as shown in Figure 7a. In Figure 7b, when the coupling gap of the 3 dB DC couplers is set as 0.2 µm, the 3 dB state (the same intensity in Port 1 and Port 2) can be realized with the coupling length *L_f_* set at 16 µm. The PIN junction structure on the one-side ridge waveguide as the EO modulation arm is defined using the ion implantation technique. The electrodes are loaded. As shown in Figure 7c, as the DC bias is above 0.8 V, the effective refractive index of the ridge waveguide as the modulation arm decreases based on the plasma-dispersion effect. When the arm length of the MZI interference is defined as 120 µm, the half-wave voltage Vπ is obtained as 1.08 V and the extinction ratio could come to 20 dB, as shown in Figure 7d. As shown in Figure 7f,g, the OPA chip designed is suitable for 50 Gbit/s transmission from the eye diagrams, which means that the modulation rate can come to 2.5 ps.

**Figure 8:**
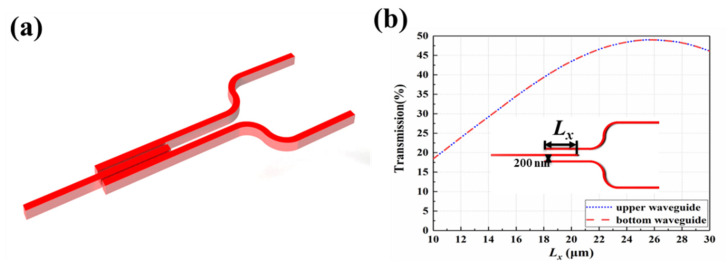
(**a**) The structure of 3-dB coupler. (**b**) The relationship between coupling length (*L_x_*) and transmission.

**Figure 9:**
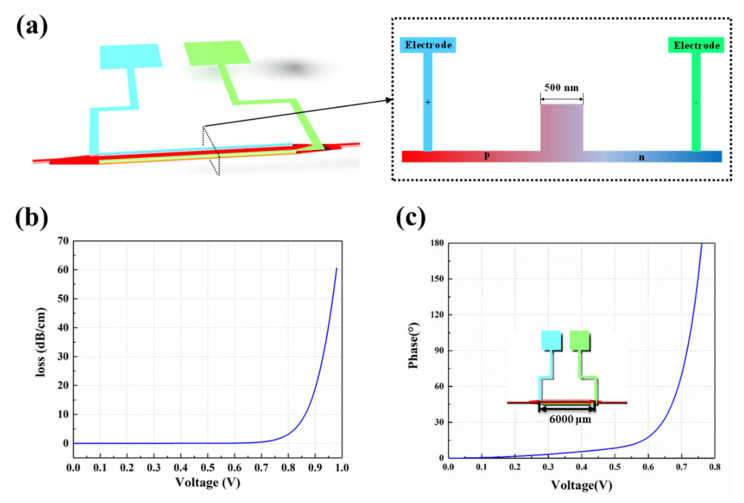
(**a**) The structure of phase shifter. (**b**) Transmission loss varies with voltage. (**c**) The relationship between phase and voltage.

The cascaded DC-MZI EO switching arrays could achieve a high-speed response of wavelength selectivity, with low consumption, and small footprint. After that, the determined signal wavelength selected from switch arrays is coupled into the 1 × 32 SOI waveguide EO phase-shifter array. The EO phase-shifter array is built by connecting 1 × 32 splitters with 32-channel EO modulation phase-shifter arrays. The equal optical phase difference is controlled using the EO phase-shifter array. To realize the uniformity of optical power distribution of broad bandwidth, the trident structure for the Y branch type splitter unit is designed as shown in Figure 8a. The coupling gap of the trident structure is set as 0.2 µm, as shown in Figure 8b; when the coupling length (*L_x_*) is 26 μm, the output intensity of each arm can come to 48%. The PIN junction waveguide structure of the phase-shifter array is given in Figure 9a. Meanwhile, the modulation of the phase shifter is analyzed at a wavelength of 1550 nm. The optical phase of the signal wavelength selected could be tuned using electrodes based on the plasma-dispersion effect. However, due to the influence of carrier absorption, when the threshold voltage exceeds 0.7 V, the absorption loss increases exponentially, as shown in Figure 9b. To inhibit the absorption loss, the length of the ridge waveguide is limited to 6 mm. Optical phase variation in the 2π range could be achieved using a driving voltage in a range from −0.76 to 0.76 V, as shown in Figure 9c. Compared to the DC-MZI EO switch, the response speed of the phase shifter will be faster than 2.5 ps due to the long modulation arm.

### 2.4. Design for Grating Emitter Arrays (Section C)

**Figure 10:**
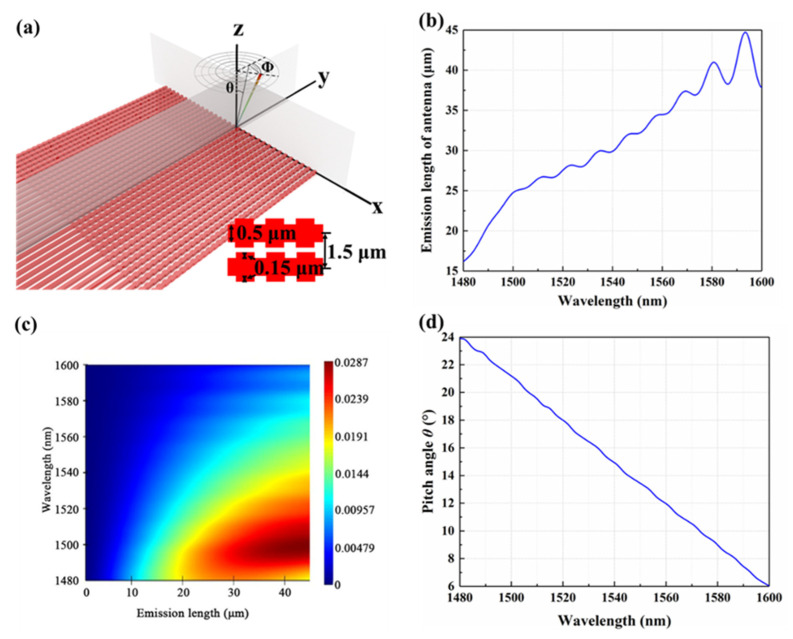
The analysis of grating antenna array: (**a**) Structure; (**b**) Relationship between emission length of antenna and wavelength; (**c**) Intensity coupled to the adjacent grating antenna; (**d**) Pitch angle (*θ*) of grating antenna in the air.

**Figure 11:**
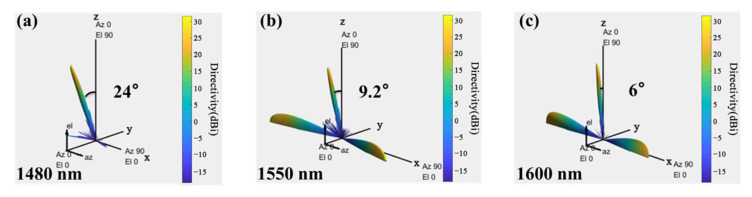
Beam formed at: (**a**) 1480 nm; (**b**) 1550 nm; (**c**) 1600 nm.

In Section C, the optical beams of the determined signal wavelength are launched from 32 channels with equal phase differences using the SOI waveguide grating antenna array. The setting data for the grating emitters are also dependent on the standardized 180 nm silicon photonic multi-project-wafer (MPW) process, provided by the Institute of Microelectronics of Chinese Academy of Sciences (IMECAS). The features of the 32-channel grating antenna array are analyzed and simulated using the finite-difference time-domain (FDTD) method. As shown in Figure 10a, the pitch angle (*θ*) and azimuth angle (*Φ*) of the beam formed by the Bragg waveguide grating array will be controlled in the polar coordinate system owing to the change in the wavelength and phase difference of the signal light, respectively. The width and ridge height of the emitting waveguide grating are set as 0.5 and 0.15 µm, respectively. The duty cycle of the Bragg waveguide grating is 50%. The gap between the adjacent grating antennas is defined as 1.5 μm, which needs 6 mm to achieve a 10% coupling efficiency. As shown in Figure 10b, the emission length required by the Bragg antenna is less than 45 μm when the wavelength ranges from 1480 nm to 1600 nm. Therefore, the grating length is set as 45 μm. The crosstalk between adjacent grating antennas with the attenuation length below 45 μm is less than 15.42 dB when the wavelength range is from 1480 to 1600 nm, as shown in Figure 10c. For 2D OPA application, as shown in Figure 10d, the pitch angle (*θ*) of the grating antenna from 6° to 24° could be achieved based on varied signal light between 1480 and 1600 nm wavelength. In addition, the pitch angle (*θ*) of the output beam increases with the decrease in the wavelength of the input signal. The beamforming of 1480 nm, 1550 nm, and 1600 nm signals are simulated using the MATLAB software, as shown in Figure 11.

**Figure 12:**
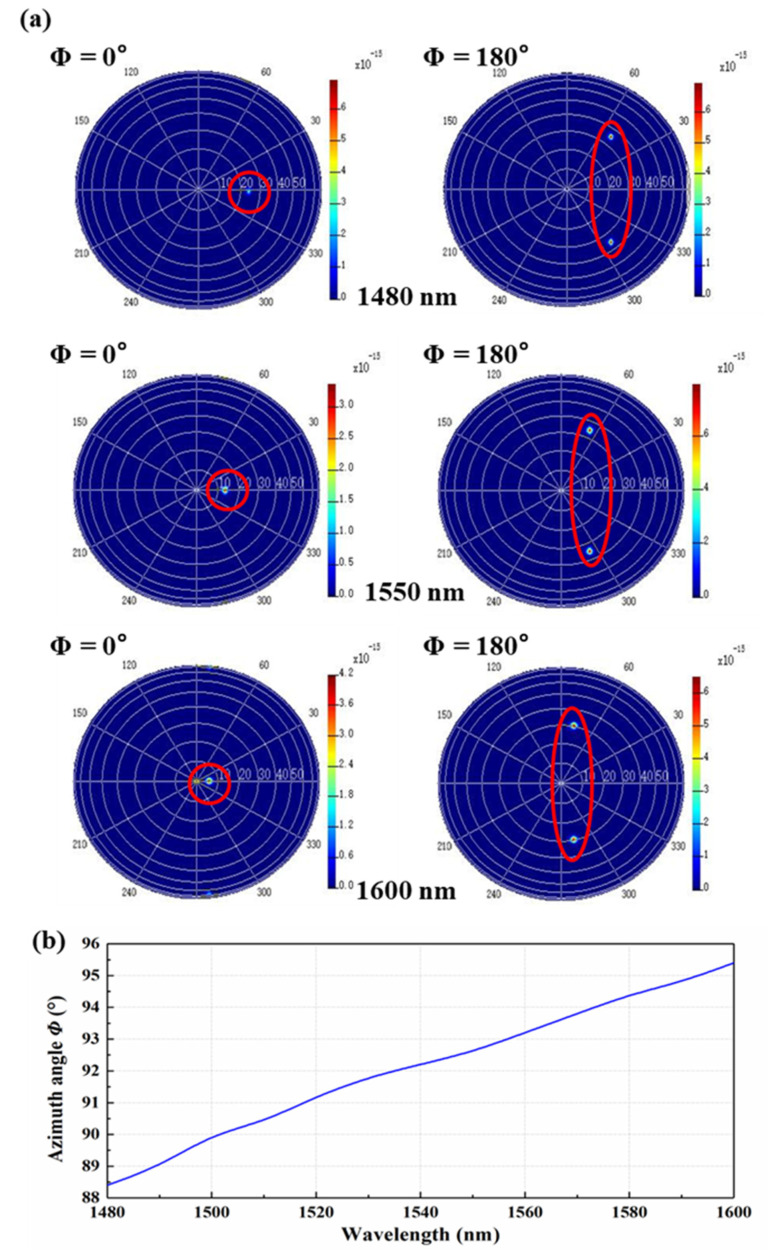
Azimuth scanning angle range analysis. (**a**)The far-field spot distribution of the OPA. (**b**) The range of the antenna azimuth scanning angles (*Φ*).

The far-field spot distribution with different bias angles is simulated using the Lumerical software. Beam synthesis with offset angles at the wavelength of 1480 nm, 1550 nm, and 1600 nm is shown in Figure 12a. There is no gate flap within ±44°, which is the azimuth angle (*Φ*) range of the OPA. The azimuth scanning angle (*Φ*) of the OPA is in the range of 88.2 to 95.4° and increases with the decrease in the input signal wavelength, as shown in Figure 12b.

As given in Table 3, compared to recently reported OPAs [3,13,27,28], the proposed monolithic 2D OPA module based on the SOI photonic platform avoids the complicated approach of connecting the external laser to the chip, and the ultra-small footprint for the OPA system designed is guaranteed. Moreover, in contrast to the TO response, the proposed modulation rate of the OPA photonic could be increased from -μs to -ps considering the EO effect.

## 3. Conclusions

In summary, monolithic 2D optical phased arrays heterogeneously integrated with on-chip laser arrays are successfully designed based on the SOI photonic platform. Rsoft and Lumerical software are used to simulate the performances of the photonic module. The use of both the software is beneficial for precisely estimating the actual features of the OPA photonic chip. The lasing power of each unit from the on-chip F-P laser arrays is more than 80 dBm. The operating voltages for controlling the pitch and azimuth angles are less than 1.1 and 1.0 V, respectively. The steering range of the pitch angle (*θ*) of the grating antenna is from 6° to 24°. The scanning range of the azimuth angle for OPA is from −44.2° to 44.2°. The overall steering range of 88.4° × 18° can be achieved by tuning both the phase and the wavelength for each antenna. The proposed modulation rate of the OPA photonic chip could come to 2.5 ps. The proposed assembly of the ultra-compact 2D OPA with on-chip tunable laser arrays using hybrid integration could be beneficial for realizing the application of a high-density, high-speed, and high-precision lidar system.

## Figures and Tables

**Table 1:** Detailed parameters for each semiconductor layer of the MQW laser.

Layer Definition	Type	Thickness	Material	Refractive Index	Carrier Concentration
P-cladding	P	1500 nm	InP	3.17	1 × 10^18^ cm^−3^
SCH barrier layer	I	40 nm	In_0.3_Ga_0.7_As_0.55_P_0.45_	3.31	
Well layer	I	*H*_1_	In_0.53_Ga_0.47_As	3.46	
Barrier layer	I	*H*_1_	In_0.3_Ga_0.7_As_0.55_P_0.45_	3.31	
Well layer	I	*H*_1_	In_0.53_Ga_0.47_As	3.46	
Barrier layer	I	*H*_1_	In_0.3_Ga_0.7_As_0.55_P_0.45_	3.31	
Well layer	I	*H*_1_	In_0.53_Ga_0.47_As	3.46	
SCH barrier layer	I	40 nm	In_0.3_Ga_0.7_As_0.55_P_0.45_	3.31	
P-cladding	P	200 nm	InP	3.17	1 × 10^18^ cm^−3^

**Table 2:** Detailed parameters for reflection gratings.

Project	Grating-I	Grating-II	Grating-III	Grating-IV
Period 1(Pd_1_) (nm)	1746.1	1802.2	1843.9	1871.9
Period 1(Pd_2_) (nm)	1760.3	1816.1	1857.9	1886.0
Period 1(Pd_3_) (nm)	1774.5	1830.0	--	1900.0
Period 1(Pd_4_) (nm)	1788.4	--	--	1914.2
The range of wavelength (nm)	1474.2–1515.5	1513.6–1545.7	1543.7–1565.6	1563.2–1605.6
Reflecting power	>99%	>99%	>99%	>99%
Working for laser array	LA-I	LA-II	LA-III	LA-IV

**Table 3:** Comparison of OPAs.

Source	Dimension	Size	Scanning Range	Type of the Phase Shifter	Modulation Rate	References
External lasers	2D	6 mm × 8 mm	104.0° × 17.6°	TO	0.67 μs	[3]
External lasers	2D	N/A	8.9° × 3.9°	TO	7 and 21 ms	[13]
External lasers	2D	3.1 mm × 1.5 mm	99.24° × 15.62°	TO	N/A	[27]
External lasers	2D	3.1 mm × 3.2 mm	6.6° × 4.4°	TO	5.7 μs	[28]
On-chip lasers	2D	8 mm × 3 mm	88.4° × 18°	EO	2.5 ps	Current study

## Data Availability

Not applicable.
